# OptORF: Optimal metabolic and regulatory perturbations for metabolic engineering of microbial strains

**DOI:** 10.1186/1752-0509-4-53

**Published:** 2010-04-28

**Authors:** Joonhoon Kim, Jennifer L Reed

**Affiliations:** 1 Department of Chemical and Biological Engineering, University of Wisconsin-Madison, Madison, WI 53706, USA; 2 DOE Great Lakes Bioenergy Research Center, University of Wisconsin-Madison, Madison, WI 53706, USA

## Abstract

**Background:**

Computational modeling and analysis of metabolic networks has been successful in metabolic engineering of microbial strains for valuable biochemical production. Limitations of currently available computational methods for metabolic engineering are that they are often based on reaction deletions rather than gene deletions and do not consider the regulatory networks that control metabolism. Due to the presence of multi-functional enzymes and isozymes, computational designs based on reaction deletions can sometimes result in strategies that are genetically complicated or infeasible. Additionally, strains might not be able to grow initially due to regulatory restrictions. To overcome these limitations, we have developed a new approach (OptORF) for identifying metabolic engineering strategies based on gene deletion and overexpression.

**Results:**

Here we propose an effective method to systematically integrate transcriptional regulatory networks and metabolic networks. This allows for the formulation of linear optimization problems that search for metabolic and/or regulatory perturbations that couple biomass and biochemical production, thus proposing adaptive evolutionary strain designs. Using genome-scale models of *Escherichia coli*, we have implemented the OptORF algorithm (which considers gene deletions and transcriptional regulation) and compared its metabolic engineering strategies for ethanol production to those found using OptKnock (which considers reaction deletions). Our results found that the reaction-based strategies often require more gene deletions to remove the identified reactions (2 more genes than reactions), and result in lethal growth phenotypes when transcriptional regulation is considered (162 out of 200 cases). Finally, we present metabolic engineering strategies for producing ethanol and higher alcohols (e.g. isobutanol) in *E. coli *using our OptORF approach. We have found common genetic modifications such as deletion of *pgi *and overexpression of *edd*, as well as chemical specific strategies for producing different alcohols.

**Conclusions:**

By taking regulatory effects into account, OptORF can propose changes such as the overexpression of metabolic genes or deletion of transcriptional factors, in addition to the deletion of metabolic genes, that may lead to faster evolutionary trajectories. While biofuel production in *E. coli *is evaluated here, the developed OptORF approach is general and can be applied to optimize the production of different compounds in other biological systems.

## Background

Metabolic engineering has emerged as an important field aimed to improve cellular production of valuable biochemicals and biofuels. Conventional approaches in metabolic engineering for identifying targets for manipulation focus on metabolic branch points, where undesired reactions are eliminated from competing branches to enhance flux through desired reactions using genetic modifications. However, these metabolic network modifications will not only affect fluxes through local metabolic pathways, but also have system-level effects on metabolic behavior due to changes in carbon, energy, and electron flow. Correspondingly, such conventional approaches may fail to identify modifications in distant pathways that can potentially improve cellular production.

Computational models of metabolism have been successful in predicting the consequences of gene deletions at a systems level [1-4]. In *Escherichia coli*, genome-scale models of metabolic networks have been used to identify metabolic engineering strategies such as gene deletions or additions to maximize production of primary or secondary metabolites [5-7]. Some computational methods, such as OptKnock [8], identify knockout strains that would have improved biochemical production capabilities after undergoing adaptive evolution. Knockout mutants that force the coupling between biomass and biochemical production allow one to use growth rate as a selective pressure and find adaptively evolved strains with improved growth rates and production capabilities. Such methods have been used to generate lactate and succinate producing strains [9,10]. A number of variations on OptKnock have appeared recently which use alternative search algorthims, add non-native pathways, and consider deviations from wildtype flux levels [5,11,12].

Computational strain design methods evaluate the effects of gene or reaction deletions to search for the mutants with improved production capabilities. A gene deletion is simulated by removing the reactions associated with the target gene from the metabolic networks; however, most current methods are often based on reaction deletions, and not gene deletions. However, genes and reactions do not always have a one-to-one relationship due to the presence of multi-functional enzymes, enzyme subunits, orphan reactions, and isozymes. Thus, knockout mutants based on reaction deletions can sometimes be genetically impossible or difficult to construct. Also, existing methods do not take into consideration the transcriptional regulatory networks that control metabolism. As a result, predicted strains with high production capabilities may not be able to grow initially or evolve to the desired final state due to regulatory restrictions.

In this study, we present a new optimization approach, OptORF, to identify metabolic engineering strategies based on a minimal number of metabolic and transcription factor gene deletions and metabolic gene overexpression, which couple biomass and biochemical production. Here, gene to protein to reaction (GPR) associations are modeled directly using a Boolean approach and reactions are removed when the associated genes are deleted. Interactions between the regulatory and metabolic networks are also modeled using Boolean approaches by turning on or off metabolic gene expression in response to transcriptional factor (TF) status. These Boolean relationships can be effectively formulated as linear constraints using binary variables and matrices, which are more systematic and/or computationally efficient than previously suggested formulations for modeling GPR associations and integrating metabolic and regulatory models [13-17].

The integrated model of metabolism and regulation can predict the steady-state metabolic flux distributions and regulatory states simultaneously. Consequently, the OptORF framework allows for the identification of optimal metabolic gene knockouts as well as transcription factor knockouts. In addition, overexpression of genes that are unexpressed under a given condition can be found in order to improve the production of a target biochemical. Using genome-scale metabolic and regulatory models of *E. coli *[18,19], we have identified metabolic engineering strategies for ethanol production using OptKnock (which considers reaction deletions) and compared these strategies to those found using our new approach OptORF (which considers gene deletions) with and without transcriptional regulatory constraints. Our analysis showed that the strategies based on reaction deletions often require a larger number of gene deletions, and also many of them result in lethal growth phenotypes when transcriptional regulation is considered. In addition, we have identified metabolic engineering strategies for overproduction of higher alcohols such as isobutanol via non-fermentative pathways based on a recent study [20]. While ethanol and higher alcohol production in *E. coli *is evaluated here, the OptORF approach can be easily applied to other biochemicals and microorganisms.

## Methods

OptORF is a bi-level optimization problem which identifies the optimal metabolic and regulatory gene deletions as well as gene overexpressions that maximize biochemical production at the maximum cellular growth under transcriptional regulatory constraints (Table 1). The inner problem of OptORF, which is a linear programming (LP) problem, maximizes growth under the given gene deletions and regulatory states that are determined by the constraints of the outer problem. OptORF is formulated as a single level mixed integer linear program (MILP) by replacing the inner maximization problem with its optimality conditions as constraints. GPR associations and transcriptional regulatory constraints are systematically formulated using three dimensional arrays, which differ from recently reported approaches [13,15,17].

**Table 1:** OptORF formulation

**maximize**	***biochemical production***	
**subject to**		
	**maximize**	***cellular growth***
	**subject to**	steady-state mass balance
		enzyme capacity
		thermodynamics
		reaction deletions
	
	GPR associations	
	transcriptional regulations	
	gene deletions and overexpressions	
	limited number of gene deletions	
	limited number of gene overexpressions	

**Figure 1:**
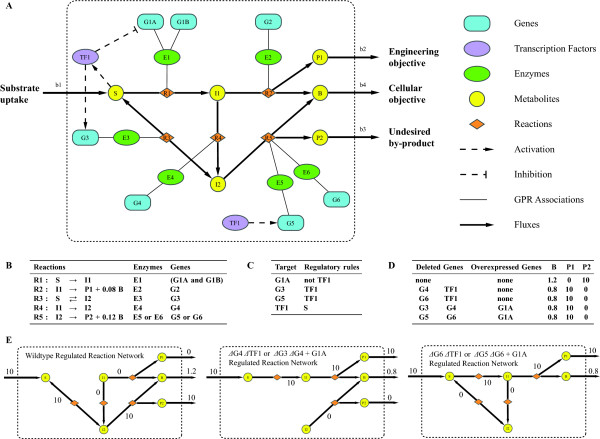
**Application of OptORF to an example metabolic and regulatory network**. (A) In this example network, substrate S is utilized to produce biomass B and by-products P1 or P2. Here, the engineering objective is the production of P1 and the cellular objective is the production of B. Since reaction R5 produces more B than reaction R2, P2 is the preferred by-product and P1 production is uncoupled to biomass production (no P1 is produced when biomass is maximized). (B) Reactions in the network and their GPR associations. (C) Transcriptional regulation of the metabolic genes and transcription factor in the network. (D) Metabolic engineering strategies for the production of P1 identified by OptORF, where P1 is now produced when biomass production is maximized in the altered networks. (E) A schematic view of the OptORF solutions. The metabolic flux distributions for wildtype and mutants are shown to describe how the optimal flux profiles (where production of biomass, B, is maximized) in the integrated network changes by the metabolic and/or regulatory perturbations presented in (D).

An example of an integrated metabolic and transcriptional regulatory network is shown in Figure 1. In this network, a substrate (S) can be utilized to produce biomass (B) via either intermediate metabolite I1 and/or I2. Reaction R2 converts I1 into by-product P1 and 0.08 B, whereas reaction R5 converts I2 into by-product P2 and 0.12 B. Reaction R1 is carried out by enzyme E1 which consists of two subunits encoded by gene G1A and G1B. Reaction R5 can be carried out by either enzyme E5 or E6, which are encoded by genes G5 and G6 respectively. Transcription factor TF1 is active when S is present, and activates expression of G3 and G5, and represses G1A expression (all other genes are considered to be expressed under all conditions in the model).

Since the cellular objective is maximizing biomass production (B) from substrate (S), pathways involving R5 (producing P2) would be normally preferred to ones involving R2 (producing P1). Given an engineering objective of producting P1, close inspection of the reaction network indicates that removal of reactions R3 and R4 or reaction R5 would couple maximum biomass production to production of P1 instead of P2. OptORF will identify genetic modification strategies involving gene deletions that are associated with these reactions (G3 and G4, or G5 and G6, respectively). However, G1A expression is inhibited by TF1, and TF1 is active in the presence of S, and thus reaction R1 cannot happen. Therefore, OptORF will also identify the overexpression of G1A along with the gene deletions mentioned above. An alternate strategy to the overexpression of G1A would be the deletion of TF1 which inhibits expression of G1A. In fact, when TF1 is deleted, the genes activated by TF1 (G3 and G5) would be no longer expressed, which reduces the number of genes that are needed to be deleted. Therefore, OptORF will first identify double knockout strategies including the TF1 deletion, and then find the alternate strategies with the G1A overexpression (these strategies are shown in Figure 1, see Additional file 1 for the implementation).

### GPR association

Constraints for GPR associations are systematically formulated using a three dimensional array (*GPR*(*j, n, g*)) and binary variables for reaction (*d*_*j*_), enzyme (*b*_*n*_), and gene (*y*_*g*_) status, where *j*, *n*, and *g *specify a reaction, enzyme, and gene, respectively. Each reaction with a known GPR association (*j *∈ *J*_*GPR*_) can be carried out by the associated enzyme complex(es) (*n *∈ *N*(*j*)), and each enzyme complex is associated to gene products (*g *∈ *G*(*n*)), where *J*_*GPR*_, *N*(*j*), *G*(*n*) are defined as the following:

If any of the enzymes for reaction *j *are present (any *b*_*n*(*j*) _= 1), the reaction can have a non-zero flux (*d*_*j *_= 1) where *d*_*j *_indicates whether a reaction can or cannot occur. If all the enzymes are not present (all *b*_*n*(*j*) _= 0), then the reaction cannot occur (*d*_*j *_= 0). This reaction-enzyme logical relationship can be formulated as the following:(1)(2)

If all of the associated genes for enzyme *n *are expressed (all *y*_*g*(*n*) _= 1), then the enzyme is present (*b*_*n *_= 1). If any of the subunits are not expressed (any *y*_*g*(*n*) _= 0), then the enzyme is not present (*b*_*n *_= 0). This enzyme-gene logical relationship can be formulated as the following:(3)(4)

Reactions without known GPR associations are not constrained by these GPR rules.

### Transcriptional regulation

Transcriptional regulation of metabolic genes are also formulated as linear constraints using a three dimensional array (*TR*(*g, m, r*)) and binary variables for gene expression/transcription factor (TF) activity (*y*_*g*_), condition (*a*_*m*_), and effector status (*x*_*r*_), where *m *specifies a condition for gene expression/TF activity and *r *affects these conditions (*m*). Effectors (*r*) can be TFs, flux values, and environmental conditions. The sets that are used in the formulation are defined as follows:

Each metabolic gene, *g *∈ *G*_*MET*_, is transcribed if the conditions for its expression, *m*(*g*), are satisfied. If any of the conditions for gene *g *expression is satisfied (any *a*_*m*(*g*) _= 1), the corresponding gene is expressed (*y*_*g *_= 1). If none of the conditions for expression are satisfied (all *a*_*m*(*g*) _= 0), then the gene is not expressed (*y*_*g *_= 0). Similarly, each transcription factor, *g *∈ *G*_*TF*_, is active (*y*_*g *_= 1) if any of the conditions for its activity is satisfied. If none of the conditions for activity are satisfied, then the transcription factor is inactive (*y*_*g *_= 0). Then, the binary variables for TF activity (*y*_*g*_) are used to constrain the binary variables for TF effectors (*x*_*g*_) which determine the conditions for metabolic gene expressions or other TF activities.(5)(6)(7)

Each condition for gene expression or transcription factor activity, *m*, has its associated effectors, *r*(*m*). If all the associated activators are active (all  = 1) and repressors are inactive (all  = 0), then the condition for gene expression or transcription factor activity is satisfied (*a*_*m *_= 1). If any of the activators are inactive (any  = 0) or repressors are active (any  = 1), the condition for gene expression or transcription factor activity is not satisfied (*a*_*m *_= 0).(8)(9)(10)

Each effector, *r*, can be a transcription factor (TF), positive metabolic flux (PF), negative metabolic flux (NF), or other environmental stimuli (ES). Intracellular and extracellular stimuli are reflected by the positive or negative metabolic flux indicators by constraints, where intracellular stimuli are dependent on the flux values (*v*_*j*_) of internal reactions and extracellular stimuli are dependent on the flux values of exchange reactions (secretion or uptake). A threshold value (*ε *= 10^-3^) is used to determine whether the flux is positive (*v*_*PF *_≥ *ε*) or negative (*v*_*NF *_≤ -*ε*) as the following:(11)(12)(13)(14)

The constraints including 'if' indicators were implemented directly using the GAMS/CPLEX indicator constraint facility instead of the Big M method. Other environmental stimuli (ES) such as oxidative stress or high osmolarity were assumed to be absent in this study.(15)

### Gene deletion and overexpression

A gene deletion or overexpression is implemented by introducing gene knockout indicators (*z*_*g*_), gene overexpression indicators (*w*_*g*_) and surrogate gene expression indicators () as the following:(16)(17)(18)

The gene knockout indicator allows an expressed gene (*y*_*g *_= 1) to be unexpressed ( = 0), and the gene overexpression indicator allows a repressed gene (*y*_*g *_= 0) to be expressed ( = 1). If an expressed gene is deleted (*y*_*g *_= *z*_*g *_= 1, and *w*_*g *_= 0), the value of its surrogate gene expression indicator is equal to zero ( = 0). If a gene is not expressed (*y*_*g *_= *w*_*g *_= 0), then the surrogate gene expression indicator assumes a value of zero ( = 0) and no gene knockout is allowed (*z*_*g *_= 0). If a repressed gene is overexpressed (*y*_*g *_= *z*_*g *_= 0, and *w*_*g *_= 1), its surrogate gene expression indicator takes a value of 1 ( = 1). No overexpression is allowed if a gene is already expressed (*y*_*g *_= 1, and *z*_*g *_= *w*_*g *_= 0), and the surrogate gene expression indicator assumes a value of 1 ( = 1). A gene can be either deleted or overexpressed, but not both at the same time. Similarly, a transcription factor deletion is implemented by allowing an active TF (*y*_*g *_= 1) to be deleted ( = 0, and *z*_*g *_= 1). However, the variables *w*_*g *_are not introduced for all TFs to prevent an inactive TF from being active. The total numbers of gene deletions and overexpressions are limited to desired values, *K*_1 _and *K*_2_, respectively.(19)(20)

Then, instead of the gene expression/TF activity indicators, the surrogate gene expression/TF activity indicators are used to determine the enzyme/TF status and thus reaction states via GPR associations and transcriptional regulation by equations (21)-(23) which replace equations (3),(4), and (7).(21)(22)(23)

### Optimality condition

The bi-level optimization problem can be formulated as an MILP using the strong duality theorem in the similar way as described in OptKnock [8]. Here, a general procedure to construct the optimality conditions for the inner LP problem is presented without using large bounds for primal and dual variables. The objective function and simulated conditions are specified using a linear combination of fluxes () and lower bounds for each flux (), respectively. The primal LP (P) is formulated as follows:(24)(25)(26)

The reversible reactions (*j *∈ *J*\*J*_*LB*_) are only constrained by the mass balance equation, and associated with these constraints are unconstrained dual variables (*u*_*i*_). The uptake, secretion, or irreversible reactions are additionally constrained by the lower bounds (), and associated with these constraints are positive dual variables (*λ*_*j*_). The reactions removed by gene knockouts are constrained to zero by using binary variables (*v*_*j *_= 0 if *d*_*j *_= 0), and associated with these constraints are unconstrained dual variables (*h*_*j*_). The dual LP (D) is formulated as follows.(27)(28)(29)(30)

At optimality, the values of the objective functions in (P) and (D) are equal, and primal and dual variables satisfy the constraints of (P) and (D), respectively. The following optimality conditions for the inner problem are always satisfied as the values of all binary variables (*d*_*j*_) are fixed to 0 or 1. The inner problem can be written as:(31)

In this study, we used the biomass formation as the objective function of inner problem (*p*_*j *_= 1 for *j *= biomass formation). The constraints including 'if' indicators are implemented directly using the GAMS/CPLEX indicator constraint facility. We also constrained the dual variables for reaction removal (*h*_*j*_) to be within a small range (-1 to 1) in order to reduce the solution time (J. Kim, J.L. Reed, and C.T. Maravelias, *in preparation*).

### OptORF formulation

The objective function in the outer problem of OptORF formulation is a linear combination of fluxes with penalty terms for the total number of gene deletions or overexpressions (). The first term defines biochemical production of interest, the second term applies a weighted penalty (*α*) to an additional gene deletion, and the third applies a penalty (*β*) to an additional overexpression. In other words, the biochemical production rate should increase at least by *α *or *β *if an additional gene is deleted or overexpressed, respectively. These penalty terms can be very useful for eliminating strains needing more genetic modifications if the improvement in production is small. When *α *or *β *is a very small value (≈ 10^-6^), it effectively eliminates unnecessary modifications from the solution without affecting the optimal biochemical production. For example, if deleting gene A results in the same product yield as deleting gene A and B (i.e. deletion of gene B does not improve the yield), then the gene B deletion would not appear in the optimal solution.

If multiple solutions are desired, integer cuts constrain successive optimal solutions with a parameter (*δ*), which is the number of differences in genes among identified strategies. A previously identified solution (*k*) is comprised of a set of gene deletions and overpressions that are stored in parameters  and , respectively. If *δ *is set to 1, integer cuts prevent OptORF from finding the same solution as the previous ones. One may set *δ *to a higher value in order to obtain a more diverse set of metabolic engineering strategies. In this study, we used *α *= 10^-6^, *β *= 10^-6^, and *δ *= 1. The complete OptORF algorithm is defined by the following equations:(32)

### Models and simulation conditions

In this study, we have implemented an integrated model of metabolism and regulation for *E. coli*, iMC1010 ^*v*2 ^[19], which consists of 906 metabolic genes and 104 TFs. There was one transcription factor, GlnL, that was included in the original model but was missing regulatory targets. GlnL should affect GlnG activity, but instead GlnG activity is independent of GlnL (the correct rule for GlnG should be (GlnL AND Not (nh4(e)>2)). However, this missing regulatory interaction would not affect the results of this study as GlnG is not active under these conditions and was not identified as a strategy for improving production of the alcohols examined here. In the OptKnock simulations, we excluded transport reactions for acetate, carbon dioxide, formate, phosphate, and water from consideration as eliminating transport may be challenging. In addition, ATP synthase deletion was excluded from consideration since the deletion resulted in a high variability in ethanol production at the predicted optimal growth condition. Equivantly, the deletion of *focA*, *focB*, and *atp *operon were excluded from the OptORF simulations. The OptORF approach was applied to identify metabolic engineering strategies for overproduction of ethanol or higher alcohols (i.e. *c*_*j *_= 1 for *j *= desired alcohol secretion) in glucose minimal media. Maximum glucose uptake rate (GUR) and oxygen uptake rate (OUR) are specified in order to simulate anaerobic growth conditions (GUR = 18.5 mmol/gDW/hr, OUR = 0 mmol/gDW/hr) [21]. A minimal growth rate was set to 0.1 hr^-1 ^for all simulations. The optimization problems were solved using CPLEX 11.2 accessed via the General Algebraic Modeling System (GAMS).

## Results and Discussion

We identified metabolic engineering strategies for ethanol production in *E. coli *using the OptORF formulation with an integrated model of metabolism and regulation, and compared the resulting strategies to ones using a previous approach based on reaction deletions (OptKnock). First, a set of reaction deletion strategies was obtained using OptKnock, and then a corresponding set of gene deletions needed to remove the reactions in each OptKnock strategy was identified. These OptKnock gene deletion designs were then compared to the gene deletion strategies found by OptORF without considering transcriptional regulation to examine the differences between the reaction-based strategies and gene-based strategies. To investigate how transcriptional regulation affects adaptive evolution of microbial strains, we analyzed available data for adaptively evolved *E. coli *mutant strains using the integrated metabolic and regulatory model. OptKnock strategies were then re-analyzed using an integrated metabolic and regulatory model and compared to the OptORF strategies identified when transcriptional regulatory constraints were considered. Finally, we present metabolic engineering strategies for overproducing ethanol or higher alcohols in *E. coli *that include both metabolic gene deletions and overexpressions, as well as transcription factor deletions, using our developed approach.

### Reaction deletion vs. gene deletion

**Figure 2:**
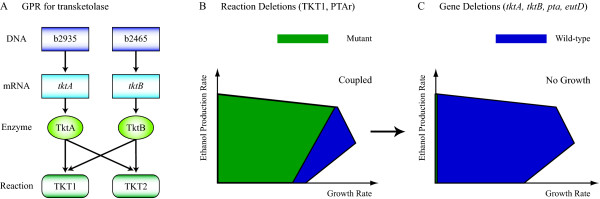
**Differences between reaction deletions and gene deletions**. (A) Gene to protein to reaction (GPR) association for transketolase reactions (TKT1 and TKT2) in *E. coli*. (B) Solution spaces for wild-type (blue) and TKT1-PTAr reaction deletion strain (green) predicted by the metabolic model. Ethanol production is coupled to cellular growth at an improved rate in the mutant strain. (C) When the corresponding genes (*tktA, tktB, pta*, and *eutD*) are deleted to eliminate TKT1 and PTAr, no growth is predicted for the mutant strain as indicated by the solution space only residing on the y-axis.

In this section, we compare reaction-based deletions to gene-based deletions and describe how the OptKnock and OptORF approaches differ in the strategies they identify. In most cases, the relationship between genes, proteins, and reactions is not one-to-one. A metabolic reaction can be carried out by one or more enzymes, each of which can be comprised of multiple gene products. An enzyme can catalyze multiple reactions that utilize different substrates, and different enzymes may catalyze the same reaction. Consequently, removal of a reaction may require deletion of multiple genes and may accompany the removal of additional reactions, which can result in a different metabolic solution space from the one predicted when reactions can be removed individually. Figure 2 shows an example, where different outcomes are found for reaction deletions and gene deletions. There are two transketolases (TktA and TktB) in *E. coli *each of which catalyzes two reactions (TKT1 and TKT2) in the pentose phosphate pathway. By removing TKT1 reaction along with phosphotransacetylase reaction (PTAr), ethanol production can be coupled to cellular growth at an improved production rate. However, deletion of the two genes (*tktA *and *tktB*) needed to remove the TKT1 reaction results in a lethal growth phenotype [22], which is correctly predicted by the model since TKT2 would also be eliminated. This illustrates how the coupling of cellular growth and biochemical production by reaction-based strategies may no longer occur when the necessary genes are deleted. Moreover, if the reaction that needs to be removed happens spontaneously or does not have known gene(s) associated to it, there is no practical way to genetically engineer the cells.

The number of genetic manipulations needed is an important factor when evaluating metabolic engineering strategies. When isozymes are present, a strategy with the minimum number of reaction deletions does not necessarily correspond to a strategy with the minimum number of gene deletions. For example, there are four gene products in *E. coli *known to function as serine deaminases. In order to completely remove this particular metabolic reaction from the system, one would have to knockout all four genes. If removal of an alternative reaction would serve the same purpose, but require fewer gene deletions, then OptORF would identify the simpler genetic strategy while reaction-based frameworks would not be able to distinguish between them.

**Figure 3:**
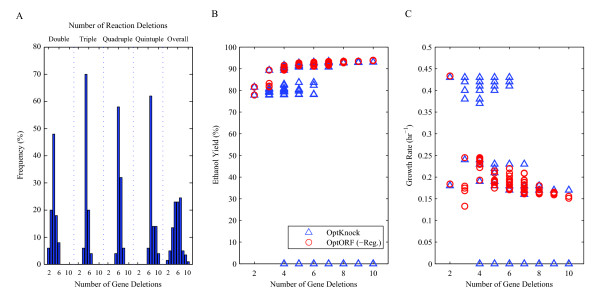
**Comparison of reaction-based deletion strategies (OptKnock) and gene-based deletion strategies (OptORF)**. (A) A total of 200 optimal reaction deletion strategies (50 double, triple, quadruple, and quintuple reaction deletions each) were found by OptKnock, and the minimal number of gene deletions that are required to remove the identified reactions were found for each OptKnock strategy. The last histogram (overall) shows the overall distribution of required gene deletions across all 200 strains. (B-C) For each OptKnock strategy, an analogous strategy was then identified with OptORF which had the same number of gene deletions as the OptKnock strategy, giving 200 OptORF strains with the same overall gene deletion distribution as shown in (A). The ethanol yields and growth rates for the 200 OptKnock strains (blue triangles) and 200 OptORF strains (red circles) are shown in panels B and C, with OptORF strains having higher ethanol yields as compared to OptKnock strains for the same number of gene deletions.

We have analyzed the top 50 ethanol producing strains found by OptKnock for each double, triple, quadruple, and quintuple reaction deletion (200 in total, see Additional file 2). Figure 3A shows the minimum number of gene deletions required to remove the identified set of reactions for each case. On average, we found that two more gene deletions would be required than the number of reaction deletions to completely eliminate the reactions suggested by OptKnock from the network (e.g. a four reaction deletion strategy would require deleting six genes). The identified set of necessary gene deletions also resulted in removal of additional reactions (which are not accounted for in OptKnock) in most of the cases (196 out of 200 cases), sometimes resulting in lethal growth phenotypes (31 out of 200 cases). For each OptKnock strategy found, we identified another strategy requiring the same number of gene deletions using OptORF without transcriptional regulatory constraints (see Additional file 2). This gave us metabolic gene deletion strategies that can be compared to ones identified by OptKnock. As shown in Figure 3B, the ethanol yields for OptORF designed strains were higher than the yields for OptKnock strains, and all OptORF strains were capable of growing (Figure 3C).

From a computational point of view, gene deletions can be more advantageous than reaction deletions due to the nature of combinatorial optimization. The difficulty of solving such an optimization problem increases exponentially with the total number of decision variables, i.e., reactions or genes to choose from. Generally, the total number of reactions are larger than the total number of genes in available genome-scale models. For example, the most recent metabolic reconstruction of *E. coli *K-12 MG1655 [23] includes 2,381 reactions, but only 1,260 ORFs are accounted for. Although OptORF requires a number of binary variables for genes, proteins, and regulatory rules, these are very tightly constrained by the GPR association and transcriptional regulatory constraints. As a result, the computation time to solve an OptORF problem is comparable to the time to solve an OptKnock problem.

### Adaptive evolution and transcriptional regulation

Transcriptional regulation plays a significant role in controlling the expression of metabolic genes thereby affecting flux through metabolic reactions. These regulatory effects have not been directly considered in previous strain design approaches. Transcription factors not only affect metabolic flux distributions by controlling gene expression, but they also sense and respond to metabolic or environmental changes. Integrating transcriptional regulatory networks with metabolic networks requires the connection between genes and reactions. We have effectively formulated these transcriptional regulatory and gene to protein to reaction (GPR) logical relationships, which enables us to predict the effects of transcription factor deletions as well as metabolic gene deletions on transcription regulation and metabolism, simultaneously.

Metabolic engineering strategies described in this work are based on the assumption that microbial cells would evolve to have higher growth rates, and that biochemical production would increase along with cellular growth rate, the latter being the selective pressure during adaptive evolutionary experiments. An important question that one might ask is how malleable the transcriptional regulatory network is during adaptive evolution. If cells can easily rewire their transcriptional networks to gain higher fitness, it is possible that knockout strains could lose the coupling of biochemical production and growth, if expressing unexpressed genes leads to a higher growth rate without a higher biochemical production. To address this issue, we have analyzed available data for adaptively evolved strains of *E. coli*, and compared the data to predictions using the integrated model of metabolism and regulation.

**Figure 4:**
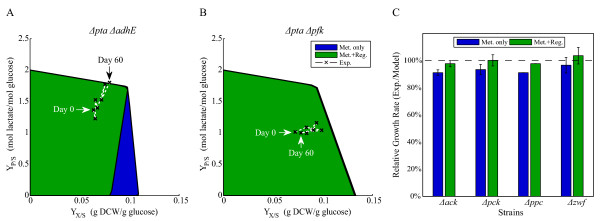
**Adaptive evolution of *E. coli *mutants**. (A-B) Adaptive evolution of lactate producing *E. coli *strains for 60 days. The data from the lactate producing strains [9] were compared to integrated metabolic and regulatory model predictions. The production capabilities for each mutant was calculated without considering transcriptional regulation (blue) and considering transcriptional regulation (green). Experimentally observed trajectories are shown for every 10 days of adaptive evolution on the model predicted solution spaces. (C) Model predictions for adaptively evolved *E. coli *strains grown on malate. Previously reported data on the adaptive evolution of single gene knockout strains [1] was compared to simulations of the gene deletion mutants using the metabolic model (blue) and the integrated metabolic and regulatory model (green). The relative growth rates (experimentally observed/model predicted) were calculated for each strain at the last day of evolution using the two models. Error bars indicate one experimental standard deviation among two or three independently evolved strains.

First, previously experimentally implemented *E. coli *strains designed for lactate production [9] were re-assessed using the integrated metabolic and regulatory models. Figure 4A and 4B show the possible lactate and biomass yields for Δ*pta*Δ*adhE *and Δ*pta*Δ*pfk *strains predicted by the metabolic model (blue) and integrated metabolic and regulatory model (green). The experimental observations for lactate yields and biomass yields during 60 days of adaptive evolution are also shown. All the deletions were simulated based on gene deletions and not reaction deletions, and yields were plotted to normalize the fluxes to changes in glucose uptake rate which occured during adaptive evolution. The Δ*pta*Δ*adhE *strain is only predicted to produce more lactate with increased growth by the integrated metabolic and regulatory model, while no coupling between lactate production and growth is predicted by either model for the Δ*pta*Δ*pfk *strain. Experimentally observed trajectories for Δ*pta*Δ*adhE *strain move towards the optimal point predicted only by the integrated metabolic and regulatory model (Figure 4A), while the Δ*pta*Δ*pfk *strain does not exhibit improved lactate production in agreement with both models (Figure 4B).

Metabolic gene deletion strains have also been evolved on different carbon sources [1]. We have analyzed growth phenotypes for these strains using the metabolic model and integrated metabolic and regulatory models, and found that only the strains grown on malate showed a significant difference in predicted growth rates between the regulated and un-regulated models. Figure 4C shows the experimentally observed growth rates relative to the predictions for mutant strains grown on malate at the end of adaptive evolution (day 40). Mutant strains seem to evolve and increase their growth rates to the optimal values predicted by the integrated model, but do not reach the values predicted by the metabolic model alone. The only strain that did exceed the integrated metabolic and regulatory model predictions, Δ*zwf*, also had large experimental standard deviations in the observed growth rates. Based on these results, it is possible that cells undergoing adaptive evolution do not significantly rewire their transcriptional regulatory networks, and therefore regulation should be considered in the design of production strains.

Another advantage of using an integrated model of metabolism and regulation emerges when it comes to predicting essential genes. An integrated model is better at predicting essential genes under a given condition, and hence more likely prevents gene deletions which are lethal from being included in the strategies. It was previously shown that an integrated model of *E. coli *correctly predicts the growth phenotypes for 10,833 (78.8%) of the total 13,750 cases (mutant grown in a single environmental condition), while a metabolic model alone predicts 8,968 (65.2%) cases correctly [19]. An integrated model is also capable of predicting essential transcription factors (e.g. *cysB *and *metR*) as well as metabolic genes in *E. coli *[19,24,25]. Accordingly, strains that are designed with regulatory considerations should grow better initially and may achieve the desired phenotype faster.

### Metabolic model vs. integrated model

**Figure 5:**
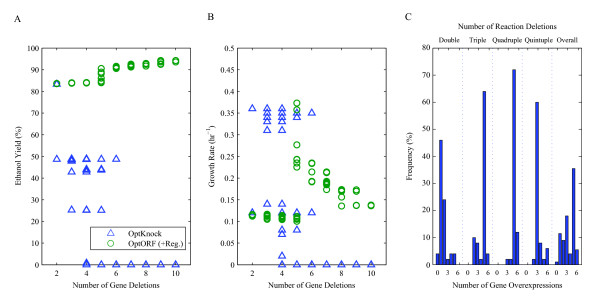
**Comparison of strategies by the metabolic model and the integrated metabolic and regulatory model**. The OptKnock deletion strategies in Figure 3 were re-analyzed using the integrated metabolic and regulatory model, where their corresponding gene deletions were the same but the maximum growth rate and ethanol production were re-calculated with the integrated model. (A-B) The ethanol yields and growth rates for most of the OptKnock strains (blue triangles) were predicted to be significantly lower by the integrated model than the those predicted by the metabolic model alone (Figure 3B and 3C) due to transcriptional regulatory constraints. Also shown are strain designed using OptORF (green circles) when transcriptional regulation is included (gene overexpression was not allowed such that K_2 _= 0). (C) For each OptKnock strategy, we identified the minimal number of genes (whose expression is repressed by the transcriptional regulation) that were needed to be overexpressed to achieve the same ethanol yield as predicted by the unregulated metabolic model (Figure 3B and 3C).

The ethanol producing strains identified in the first result section were re-analyzed using the integrated metabolic and regulatory model to demonstrate the differences from using the metabolic model. When we re-calculated the production rates and growth rates for the 200 previously identified OptKnock strains after imposing regulatory constraints, we found that ethanol production was significantly lower for most strains (Figure 5A, see Additional file 2). This is attributed to the fact that some of the regulated enzymes are not being expressed according to the transcriptional regulatory constraints. For each OptKnock strain we subsequently identified the minimum number of genes that need to be overexpressed to achieve the same ethanol yields when regulatory affects are not considered (Figure 5C, see Additional file 2). In other words, we found the sets of genes which are down-regulated by transcriptional regulation, but are necessary to achieve the same growth and ethanol production rates as shown in Figure 3. Without overexpression of these genes, the ethanol yields of OptKnock strains were much lower than the yields of OptORF strains identified when regulatory effects are considered in the strain design process (Figure 5A). Also, the number of lethal growth phenotypes for OptKnock strains were much higher when regulation is accounted for (162 out of 200 cases) implying that these strains would not be able to grow, at least initially (Figure 5B), and would possibly be difficult to construct. Interestingly, OptORF strains exhibit a sharp increase in growth and ethanol yields between 4 and 5 gene deletion strategies. This is due to the expression of genes involved in the Entner-Doudoroff pathway, and this pattern was not observed in Figure 3B and 3C when regulatory interactions were not considered in the design of OptORF strains.

**Figure 6:**
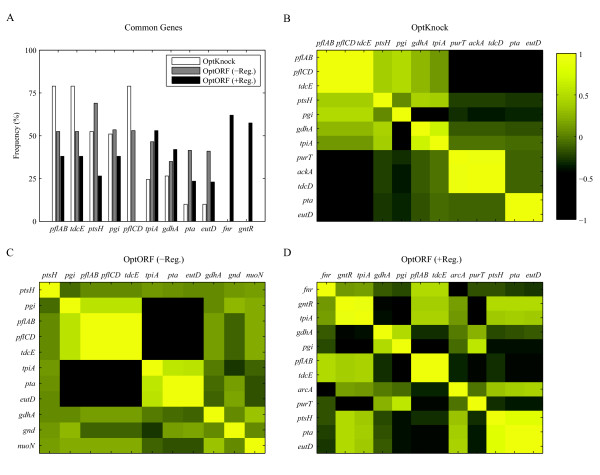
**Commonly found gene deletions for ethanol production and their correlations**. (A) The histogram shows the frequency of the gene deletions that appear in at least 15% of all 600 strategies. (B-D) For each approach, a correlation heat map for the genes that appear in at least 10% of the 200 strategies is shown. If there is a strong positive (negative) correlation between a pair of genes, the corresponding cell is colored as yellow (black).

Finally, the strain designs identified by OptKnock (200), OptORF without regulatory constraints (200), and OptORF with regulatory constraints (200) were compared to identify common genetic strategies. Figure 6A shows the list of commonly found gene deletions for different approaches and their frequency. Among the top 200 strategies found by each approach (shown in Figures 3 and 5), genes that appear in at least 15% of the total 600 strategies are listed. Overall, deletion of pyruvate formate-lyase (PFL) was the most frequent for all of the approaches. Deletion of phosphoenolpyruvate:sugar phosphotransferase system (PTS) was mainly found in approaches without transcriptional regulation, while the two transcription factor deletions (*fnr *and *gntR*) are identified only by OptORF when transcriptional regulation is accounted for. Deletion of *pgi *or *tpiA *was evenly distributed across all methods, indicating that one of them is typically necessary to couple growth to ethanol production (see next section for further discussion).

For each of the three approaches, we generated a heat map based on the correlation coefficients between the genes that appear in at least 10% of their corresponding 200 strategies (Figure 6B-D). If a pair of gene deletions always appears in strategies together, the corresponding cell in the heat map is colored in yellow. A cell is colored in black when a pair of gene deletions are anti-correlated. For example, *pta *and *eutD *appear together since the deletion of both is required to eliminate the phosphate acetyltransferase activity, while either *fnr *or *arcA *appears since the deletion of either transcription factor results in a similar phenotype. The pattern of correlation becomes clearer (strategies have less variation) as the structure of the model gets simpler from a gene-based deletion with transcriptional regulation (Figure 6D) to reaction-based deletion without transcriptional regulation (Figure 6B). This indicates that as models account for the complex structure and interactions of networks, more diverse metabolic engineering strategies can be identified.

### Strain designs for ethanol production by OptORF

We have employed OptORF to identify metabolic engineering strategies for ethanol production in *E. coli*. Strains are designed to grow on glucose minimal media in anaerobic conditions. Notable differences from the previously reported strain designs [12] are that these strategies include the deletion of transcription factors (e.g. Fnr, ArcA, or GntR) and electron transport chain components (e.g. NDH-1) as well as overexpression of metabolic genes (e.g. *edd *or *fbp*). We have identified a set of strategies consisting of only gene deletions (Table 2), and another set of strategies that also include overexpression of genes (Table 3). It should be noted that the deletion of lactate dehydrogenase (Ldh) is not required in the presented strategies because ethanol production is preferred over lactate production at the optimal growth condition, and so deletion of Ldh would not be required. Lactate could be produced initially when cells are growing sub-optimally (which is probably why other studies deleted Ldh [26,27]), but lactate production would be predicted to decrease as cells adaptively evolve to higher growth rates, which favor ethanol production due to differences in redox requirements (ethanol production consumes more NADH).

**Table 2:** Gene deletion strategies for ethanol production in *E. coli*

Deleted Genes					Growth Rate (hr^-1^)	Ethanol Yield (%)
none (wild-type)					0.467	39.3
*arcA*	*pgi*				0.122	83.5
*nuoN*	*pgi*				0.121	83.6
*fnr*	*gntR*	*pflB*	*tdcE*	*pgi*	0.225	86.2
*arcA*	*gntR*	*pta*	*eutD*	*tpiA*	0.244	89.3
*fnr*	*gntR*	*pflB*	*tdcE*	*tpiA*	0.235	90.5

The predicted ethanol yields and growth rates are listed for two-deletion strategies and five-deletion strategies. Three or four deletion strategies are not shown because the ethanol yield was not significantly improved over two deletion strategies. The variability in ethanol yield at the predicted maximum growth rate was zero or very small (< 0.01%) for all cases. The ethanol yield is reported as % of the maximum theoretical yield (100% is 0.51 g ethanol/g glucose or 2 mol ethanol/mol glucose). Maximum glucose uptake rate of 18.5 mmol/gDW/hr was used to simulate anaerobic growth conditions.

**Table 3:** Gene deletion and overexpression strategies for ethanol production in *E. coli*.

Deleted Genes					Overexpressed Genes	Growth Rate (hr^-1^)	Ethanol Yield (%)
*fnr*	*pflB*	*tdcE*	*pgi*		*edd*	0.225	86.2
*fnr*	*pflB*	*tdcE*	*pgi*	*ptsH*	*edd* *fbp*	0.182	90.4
*fnr*	*pflB*	*tdcE*	*tpiA*		*edd*	0.235	90.5
*fnr*	*pflB*	*tdcE*	*tpiA*	*gdhA*	*edd*	0.214	91.4
*arcA*	*pta*	*eutD*	*tpiA*	*ptsH*	*edd*	0.192	91.6

In addition to gene deletions, overexpressed genes are identified to further improve ethanol production. The maximum number of overexpressed genes was limited to two for all cases, and strategies including less than four gene deletions are not shown due to the negligible increase in ethanol yields by gene overexpression.

Deletion of *fnr *or *arcA *is found in most strain designs, where some enzymes involved in aerobic metabolism (that are repressed by Fnr and/or ArcA) can be advantageous for ethanol production. Aerobic genes in central metabolism that are repressed by these anaerobic regulators include *aceAB, aceEF, lpd, mdh, sucAB*, and *sdhABCD*. The de-repression of malate dehydrogenase (*mdh*) was predicted to be especially important based on comparisons between flux distributions with and without *mdh*. If necessary, such repressed genes may be overexpressed, as an alternative to deleting *fnr *or *arcA *to ensure that metabolic activity is high enough to achieve the desired level of ethanol production.

Genes involved in the electron transfer chain were also identified as needing to be deleted to limit the amount of NADH oxidized by this pathway. NADH:ubiquinone oxidoreductase (NDH) I and II catalyze the transfer of electrons from NADH to the quinone pool, and the electrons are passed to fumarate by fumarate reductase (FRD), an essential enzyme for anaerobic growth. OptORF identified the deletion of NDH-1 (*nuo*), the predominant NDH under anaerobic conditions, to block electron transfer from NADH to fumarate. As a result, the model predicts a decrease in FRD flux and reduced succinate production in NDH-1 deficient strains, while flux through fumarase and malic enzyme is increased.

Deletion of *pgi *was also found in many of the strain designs for ethanol production, suggesting re-direction of flux through glycolysis to the pentose phosphate (PP) pathway or Entner-Doudoroff (ED) pathway. This increases generation of NADPH whose electrons are passed to NAD via NADH transhydrogenase, and the additional NADH is used to reduce acetyl-CoA to ethanol by alcohol dehydrogenase (AdhE). While increasing the amount of NADH available to produce ethanol, the *pgi *deletion also lowers the net ATP production and thus decreases growth rate as compared to the wild-type strain. The ED pathway consists of two enzymes, Edd and Eda, and the expression of *edd *is repressed by the transcription factor GntR. Deletion of *gntR *would de-repress the expression of *edd*, which allows for the conversion of glucose to pyruvate and glyceraldehyde-3-phosphate. Equivalently, overexpression of *edd *was identified as an alternative to deletion of *gntR*. The activation of the ED pathway in a *pgi *mutant also leads to a significant increase in growth rate, which would be favorable for industrial-scale ethanol production.

There are three enzymes, PflAB, PflCD and TdcE, which possibly function as pyruvate formate-lyase (PFL). The regulatory model indicates that expression of *pflD *requires either ArcA or Fnr as activators, and a previous study showed that PFL activity was still detected in *pflA *or *pflB *mutant [28]. Another study revealed that a *fnr *deletion alone is sufficient to decrease PFL activity down to the level of *ΔfnrΔarcA *strain, while an *arcA *deletion alone did not decrease PFL activity [29]. Thus, deletion of *fnr*, *pflB*, and *tdcE *would abolish PFL activity and require cells to use pyruvate dehydrogenase (PDH) [26,30], whose expression is repressed by Fnr and ArcA in anaerobic conditions. Deletion of *fnr *would lower PFL activity and attenuate the repression of PDH, the result being the production of NADH instead of formate when pyruvate is converted to acetyl-CoA. In the absence of oxygen, some of the acetyl-CoA would be reduced to ethanol consuming two NADH molecules to maintain a redox balance.

Deletion of *pta *and *eutD *(both catalyze the conversion of acetyl-CoA to acetylphosphate) would reduce acetate production, and hence increase formation of other by-products such as ethanol, lactate, or succinate. However, multiple studies have shown that the mutations in the *ack-pta *pathway cause accumulation of pyruvate [31-33], and the integrated metabolic and regulatory model predicts the secretion of pyruvate (64% mol pyruvate/mol glucose) in a *ΔptaΔeutD *mutant. Pyruvate can be either secreted or reduced to form other fermentation by-products, but there is not enough NADH available to ferment all the pyruvate generated by glycolysis. In order to convert pyruvate to ethanol, *arcA *and *gntR *deletions are needed to derepress PDH and the ED pathway, along with a *pgi *or *tpiA *deletion to re-direct flux from glycolysis to the ED pathway. A *ΔtpiA *mutant alone could cause methylglyoxal accumulation and inhibit the anaerobic growth [34], but re-directing flux to the ED pathway should prevent methylglyoxal accumulation.

In the strategies that include both gene deletion and gene overexpression, we found that overexpression of *edd *replaced the *gntR *deletion in most strains to activate the ED pathway. In addition, overexpression of fructose-1,6-bisphosphatase (*fbp*) was predicted to increase the amount of fructose-6-phosphate, and reverse the direction of the non-oxidative branch of the PP pathway in the strains utilizing the ED pathway. The reversed PP pathway results in a decreased flux in the TCA cycle and an increased flux in the ED pathway and PDH, leading to improved ethanol production. The model predicts that *ptsH *deletion (in addition to other modifications) increases flux through the lower half of glycolysis and decreases succinate production. Switching glucose transport from the phosphoenolpyruvate:sugar phosphotransferase system (PTS) to proton symport has been shown to improve overall performance and production yield for ethanol as well as other compounds [35].

Glutamate can be synthesized via multiple pathways depending on the availability of nitrogen sources. When ammonia is abundant, an ATP-independent pathway functions to save energy by converting *α*-ketoglutarate to glutamate using NADPH. This pathway is encoded by glutamate dehydrogenase (*gdhA*), the deletion of which would require cells to use the ATP-dependent pathway that normally operates when the concentration of ammonia is low [36]. This ATP-dependent pathway would decrease growth rate, but increase the flux through the ED pathway and PDH, and improve the ethanol production.

**Figure 7:**
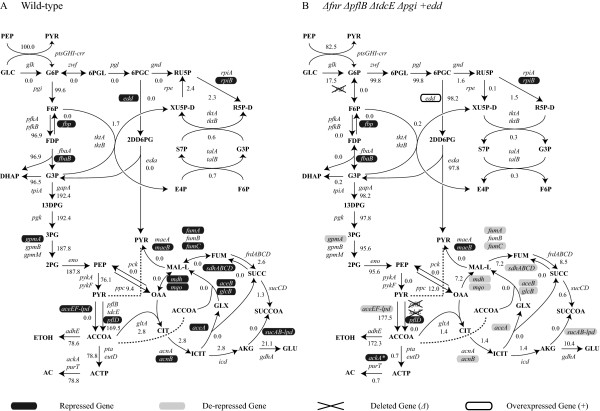
**Central metabolic state of wild-type and ethanol production strains**. Genes associated with each reaction in the central metabolic networks are shown. If isozymes exist, the corresponding genes for each isozyme are listed on separate lines. If an enzyme consists of multiple subunits, the associated genes are listed at the same line. (A) Metabolic flux distribution for wild-type strain as predicted by the integrated metabolic and regulatory model. Genes repressed by transcriptional regulation, as predicted by the integrated model, are highlighted in black. (B) Metabolic flux distribution for the *ΔfnrΔpflBΔtdcEΔpgi*+*edd *ethanol production strain as predicted by the integrated metabolic and regulatory model. Genes de-repressed by the deletion of the Fnr transcription factor are highlighted in gray. *The expression of *ackA *is not activated by Fnr in the mutant strain.

Predicted flux distributions corresponding to maximum biomass production and gene expression states are shown in Figure 7 for both the wild-type and *ΔfnrΔpflBΔtdcEΔpgi+edd *strain. These were predicted by the integrated metabolic and regulatory model (*Δ*: deletion, +: overexpression). The ethanol production rate was predicted to be approximately 86.2% of the maximum theoretical yield and the corresponding growth rate was 0.225 h^-1 ^for the mutant strain (39.3% and 0.467 h^-1 ^for the wild-type). This result is somewhat similar to the previously reported values [12], but the perturbation strategy identified by OptORF takes into account transcriptional regulatory effects, and as such could facilitate the adaptive evolution process of the mutant strain to achieve the desired phenotype. Some of the gene deletions presented here have been used previously to engineer un-evolved and evolved strains of *E. coli *for ethanol production [26,37]. The strength of the OptORF appoach emerges when these individual modifications are put together in a cooperative manner to generate a strategy, which simultaneously considers the metabolic and transcriptional regulatory network.

### Strain designs for higher alcohol production

In addition to ethanol, we have also identified metabolic engineering strategies using OptORF for over-production of higher alcohols such as isobutanol and 2-phenylethanol from glucose. Since *E. coli *does not naturally produce these higher alcohols, we have augmented the iMC1010^v2 ^network with non-fermentative reactions and corresponding GPR associations for synthesis of these alcohols based on a recent study [20]. In summary, 2-keto acid decarboxylase (KDC) and alcohol dehydrogenase (ADH) were added to the network to allow for production of 1-propanol, 1-butanol, isobutanol, 2-methyl-1-butanol, 3-methyl-1-butanol, and 2-phenylethanol from intermediates in isoleucine, leucine, and valine biosynthesis. We have assumed these enzymes, KDC and ADH, have no substrate specificity so that the production of any higher alcohol is equally preferred.

Our computational results found that most of the strategies for ethanol production can also be modified for the production of some higher alcohols with the additional deletion of AdhE (which produces ethanol) and addition of KDC and ADH. In particular, we have found that the anaerobic production of isobutanol can be coupled to growth at ~94% of the theoretical maximum yield (Table 4). In addition to the strategies found in the ethanol case, deletion of the AB-specific pyridine nucleotide transhydrogenase (*pntAB*) was identified to be beneficial for increasing isobutanol production. This prevents the electron transfer from NADH to NADP, and thus more NADH would be available for the production of isobutanol.

**Table 4:** Gene deletion and overexpression strategies for isobutanol production in *E. coli*.

Deleted Genes			Overexpressed Genes	Growth Rate (hr^-1^)	Isobutanol Yield (%)
*adhE*	*gdhA*			0.223	89.5
*adhE*	*gntR*	*pgi*		0.128	93.8
*adhE*	*pgi*		*edd* *fbp*	0.128	94.3
*adhE*	*pntA*	*nuoN*	*edd* *fbp*	0.110	95.1
*adhE*	*pntA*	*gdhA*	*edd* *fbp*	0.102	95.5

The predicted isobutanol yields and growth rates are listed for gene deletion and overexpression strategies. The two enzymes needed for synthesis of isobutanol (KDC and ADH) were assumed to be present for all cases. The variability in isobutanol yield at the predicted maximum growth rate was zero or very small (< 0.01%) for all cases. The isobutanol yield is reported as % of the maximum theoretical yield (100% is 0.41 g isobutanol/g glucose or 1 mol isobutanol/mol glucose). The simulation conditions were the same as the ethanol case.

In addition to isobutanol, we found that the production of 1-propanol and 2-phenylethanol can also be coupled to growth, but their yields were much lower than the isobutanol yield (~38% and ~5.7%, respectively, see Additional file 3). However, the production of other branched-alcohols such as 2-methyl-1-butanol or 3-methyl-1-butanol can be accompanied with the production of other alcohols including ethanol and isobutanol. In other words, cells could either produce 2-methyl-1-butanol (3-methyl-1-butanol) along with the other alcohols or produce only the other alcohols. In such cases, changes in the substrate specificity of KDC or ADH enzymes would be needed to generate specific alcohols. Interestingly, the identified metabolic engineering strategies for 2-phenylethanol production were very distinct from the strategies for other alcohol production strains (see Additional file 3). While strategies for producing other alcohols involved increasing fluxes in the oxidative branch of PP pathway and ED pathway, the strategies for 2-phenylethanol include deletion of genes in the both the oxidative (*zwf *or *gnd*) and non-oxidative (*talAB*) branches of the PP pathway. The model predicts that these gene deletions would increase the fluxes in the aromatic amino acid biosynthesis pathways, which leads to the increased availability of phenylpyruvate, the precursor for 2-phenylethanol. Analysis of these higher alcohols illustrates how OptORF can be used to couple biomass and production of metabolites which are not part of central metabolism.

## Conclusions

We have systematically integrated metabolic and regulatory models, and developed a new computational framework (OptORF) for designing microbial strains for metabolite production. We compared our new approach to OptKnock, and found four primary differences between the strains that are identified using the two approaches. First, OptKnock may propose removing reactions that do not have any genes associated with them, making the construction of such strains experimentally impossible. Second, OptORF can find metabolic engineering strategies requiring the smallest number of gene deletions while still achieving high production yields. Since OptKnock strategies are based on reaction deletions they often require more gene deletions than those found using OptORF. Third, OptKnock may suggest reaction deletions that result in a different solution space when the necessary genes are deleted or transcriptional regulatory effects are accounted for. In this case the adaptive evolutionary outcome would be different than what is predicted when only reaction deletions are considered, sometimes resulting in reduced production yields or lethal phenotypes. Lastly, OptORF can propose changes such as the overexpression of metabolic genes or deletion of transcriptional factors that may lead to faster evolutionary trajectories.

Based on our analysis of experimental data using integrated metabolic and regulatory model it is unclear to what extent, if any, cells re-wire their transcriptional regulatory network during adaptive evolution. Given that a finite number of mutations are found in adaptively evolved strains [38], it seems likely that cells could get stuck in a local maxima in the fitness landscape, where they would need to change the regulation of multiple gene products to improve fitness. This idea is supported by the fact that the same starting strain can evolve to different end points, and in some cases achieve only sub-optimal behaviors [9,39,40]. By taking regulatory effects into account when designing strains it may be possible to start with strains that are already expressing the necessary enzymes needed to achieve the desired production and growth rates. Some evolved strains may stay within the solution space defined by metabolic and regulatory constraints, while others may alter their regulatory networks if it results in a significant growth advantage, thus altering the solution space in which they evolve. Thus, it will be particularly important to conduct parallel evolutionary experiments to find evolved strains that lead to higher production without violating regulatory constraints.

In its current implementation, OptORF uses Boolean approximations to describe how transcriptional regulation affects metabolic fluxes. Although the use of Boolean variables do not exactly represent the dynamic nature of metabolism and regulation, it has been previously shown that constraint-based models using these approximations successfully predict the cellular behavior in continuous and batch culture [1,19,21,24]. The approach could be extended to include other types of regulatory models which can account for varying levels of gene expression or enzyme activity. A previous study has shown that the behavior of a transcriptional regulatory network can be well approximated by a system of linear equations near a steady-state, where gene expression does not substantially change [41]. The OptORF approach could be improved by applying these linear approximations in the regulatory part of the model, in order to describe varying gene expression levels, and using approaches to constrain metabolic fluxes based on predicted gene expression levels [42-44].

The OptORF approach is currently applied to produce metabolites that can be coupled to biomass production. A recent study has used a genetic algorithm to design strains with un-coupled metabolite and biomass production, where a bi-level problem is used and the inner problem uses an objective function to predict un-evolved cellular phenotypes [45]. OptORF could also be extended to find metabolic engineering strategies that do not require coupling of cellular growth and product formation, and would evaluate gene deletions, gene overexpression, and regulatory effects simultaneously to identify such strategies.

The novelty of the method developed here is that it accounts for transcriptional regulatory networks in addition to metabolism in the design of strains for metabolic engineering. However if desired, the approach can be used with and without transcriptional regulatory constraints to consider the interdependence of reactions through their GPR associations. It should be noted that the integrated model of metabolism and regulation allows for simulating the effects of both gene overexpression (where un-expressed genes are expressed) and gene deletion. The OptORF approach can also suggest transcription factor deletion as an alternative to metabolic gene deletion or overexpression, which provides greater flexibility in metabolic engineering strategies. By further incorporating flux modulation approaches such as those proposed in OptReg [12], additional engineering strategies can be designed which consider adjustment of flux values and not just the complete removal/addition of reactions via gene deletion or gene overexpression.

The approach we have developed here is general and can be used to engineer production of a variety of products in different microorganisms, for which constraint-based models exist. The number of microbial transcriptional regulatory network models continues to grow, which has been enabled by high-throughput datasets and computational analysis [46-52]. Regulatory networks reconstructed from analysis of high-throughput datasets can be integrated with metabolic networks using Boolean or other types of regulatory modeling formalisms, and our approach can applied to new integrated models of metabolism and regulation. As such, it will have impacts on the biological production of a wide variety of products, ranging from biofuels and other commodity chemicals to specialty chemicals [53-55].

## Authors' contributions

JK implemented the model and approach, performed the analysis, and drafted the manuscript. JLR conceived of the study, participated in its design and coordination, and helped to analyze the data and draft the manuscript. All authors read and approved the final manuscript.

## Supplementary Material

Additional file 1 **Implementation of OptORF for the example network**. The OptORF approach is implemented in the General Algebraic Modeling System (GAMS). A free demo version of GAMS can be downloaded from http://www.gams.com. This file contains the example network described in Figure 1. Click here for file

Additional file 2 **List of strain designs for ethanol production**. This spreadsheet contains the list of strain designs described in Figures 3 and 5, and corresponding growth rates and ethanol production yields. Click here for file

Additional file 3 **List of strain designs for higher alcohol production**. This spreadsheet contains the list of strain designs for 1-propanol, isobutanol, 2-methyl-1-butanol, 3-methyl-1-butanol, and 2-phenylethanol. Click here for file
